# Mapping Neurotransmitter Identity in the Whole-Mount *Drosophila* Brain Using Multiplex High-Throughput Fluorescence *in Situ* Hybridization

**DOI:** 10.1534/genetics.118.301749

**Published:** 2018-12-18

**Authors:** Geoffrey W. Meissner, Aljoscha Nern, Robert H. Singer, Allan M. Wong, Oz Malkesman, Xi Long

**Affiliations:** * Janelia Research Campus, Howard Hughes Medical Institute, Ashburn, Virginia 20147; † Department of Anatomy and Structural Biology, Albert Einstein College of Medicine, Bronx, New York 10461; † Dominick P. Purpura Department of Neuroscience, Albert Einstein College of Medicine, Bronx, New York 10461; § Gruss Lipper Biophotonics Center, Albert Einstein College of Medicine, Bronx, New York 10461

**Keywords:** neurotransmitter, *Drosophila*, fluorescence *in situ* hybridization, gene expression, mRNA

## Abstract

Identifying the neurotransmitters used by specific neurons is a critical step in understanding the function of neural circuits. However, methods for the consistent and efficient detection of neurotransmitter markers remain limited. Fluorescence *in situ* hybridization (FISH) enables direct labeling of type-specific mRNA in neurons. Recent advances in FISH allow this technique to be carried out in intact tissue samples such as whole-mount *Drosophila melanogaster* brains. Here, we present a FISH platform for high-throughput detection of eight common neurotransmitter phenotypes in *Drosophila* brains. We greatly increase FISH throughput by processing samples mounted on coverslips and optimizing fluorophore choice for each probe to facilitate multiplexing. As application examples, we demonstrate cases of neurotransmitter coexpression, reveal neurotransmitter phenotypes of specific cell types, and explore the onset of neurotransmitter expression in the developing optic lobe. Beyond neurotransmitter markers, our protocols can in principle be used for large-scale FISH detection of any mRNA in whole-mount fly brains.

A critical step in understanding the function of neural circuits is to identify the neurotransmitters used by specific neurons. Typical indicators of transmitter phenotypes are genes with specific roles in transmitter synthesis, vesicular transport, or recycling. While such markers are known for common transmitters, their reliable detection in specific cell types remains challenging, in particular if a large number of specimens need to be examined.

Common methods for detecting neurotransmitter markers are sequencing transcriptomics, immunolabeling, and fluorescent *in situ* hybridization (FISH). RNA sequencing (RNAseq)-based methods can provide comprehensive catalogs of gene expression including of neurotransmitter markers (Henry *et al.* 2012; Konstantinides *et al.* 2015; Croset *et al.* 2018; Davie *et al.* 2018; Davis *et al.* 2018). However, these approaches either examine dissociated single cells, which can be difficult to map to specific cell types, or depend on genetic markers, which are not always available, to isolate neuronal populations. In addition, both single-cell and population transcriptomics result in the loss of spatial information (Buxbaum *et al.* 2015; Lein *et al.* 2017) and incur high costs if examining a small number of mRNAs across many conditions. Immunohistochemistry preserves spatial information and can be applied on a large scale. However, high-quality antibodies are often not readily available and antibody generation can be time-consuming (Fritschy 2008). In addition, proteins that primarily localize to fine neurites may be difficult to assign to specific cells by immunolabeling.

FISH is a powerful method for detecting endogenous mRNA sequences in intact tissues (Zhao *et al.* 2003; Lécuyer *et al.* 2007; Raj *et al.* 2008; Moffitt *et al.* 2016; Shah *et al.* 2016; Yang *et al.* 2017). Recent improvements enable the localization of mRNA, including of indicators of neurotransmitter phenotypes, in whole-mount *Drosophila* tissues (Long *et al.* 2017). However, validated FISH probes are only available for marking neurons expressing acetylcholine, glutamate, and γ-aminobutyric acid (GABA), and current FISH protocols are not efficient for processing large numbers of specimens. Here, we extend detection to dopaminergic, serotoninergic, tyraminergic, octopaminergic, and histaminergic neurons with validated FISH probes, and describe a high-throughput, optimized FISH procedure for detecting these neurotransmitter cell types. The approach is particularly suitable for rapidly identifying neurotransmitter markers expressed by neurons labeled by libraries of genetic markers (*e.g.*, GAL4 or LexA driver collections). To demonstrate the utility of our FISH platform, we map neurotransmitter markers to specific cell types using split-GAL4 lines and examine the onset of neurotransmitter expression in the developing fly visual system.

## Materials and Methods

### Fly stocks

*TH-GAL4* was from Friggi-Grelin *et al.* (2003). *Tdc2-GAL4* was from Cole *et al.* (2005). *SerT-GFP* was *SerT^MI02578^* from Nagarkar-Jaiswal *et al.* (2015). *Hdc^Jk910^* was from Burg *et al.* (1993). *UAS-7xHaloTag*::*CAAX* in *VK00005* for Figure 2 and Figure 3, and Supplemental Material, Figure S6 were from Sutcliffe *et al.* (2017). UAS-myr-HaloTag for Figure S3 was from Kohl *et al.* (2014). *R58E02-GAL4* was from Liu *et al.* (2012). *SS02425* was from Davie *et al.* (2018). Wild-type flies were Canton-S. Split-GAL4 stock *SS02565* consists of *R55C09-p65ADZp* in *VK00027* and *VT040566-ZpGDBD* in *attP2* (Luan *et al.* 2006; Pfeiffer *et al.* 2010; Dionne *et al.* 2018; Tirian and Dickson 2018). Split-GAL4 stock *SS45407* consists of *VT012639-p65ADZp* in *attP40* and *VT000608-ZpGdbd* in *attP2*. Split-GAL4 stock *SS51118* consists of *VT050405-p65ADZp* in *attP40* and *VT007068-ZpGDBD* in *attP2*.

### CNS preparation

To label cells with HaloTag ligand, specific GAL4 driver lines were crossed to *UAS-HaloTag*. Flies were reared on standard corn meal molasses food at 22–25°. Approximately 3–5-day-old adult flies were used for the studies, except for the optic lobe developmental samples, for which tissues were collected at the specified developmental stages. Dissection was carried out in phosphate-buffered saline (PBS) or cold S2 medium (Schneider’s Insect Medium, S01416; Sigma [Sigma Chemical], St. Louis, MO). After dissection, brain tissues were transferred to 2% paraformaldehyde in S2 medium. Samples underwent fixation followed by one to four 15-min washes in PBS + 0.5% Triton X-100 (PBT), then were labeled with 2 μM HaloTag ligand in PBT for 15 min. Samples were washed twice in PBT then dehydrated in a 30, 50, 75, and 100% ethanol series. Samples can be stored in 100% EtOH at 4° for up to 2 weeks. HaloTag ligands were fused to either JF646 (Grimm *et al.* 2015), AF488 (G1001; Promega, Madison, WI), or ATTO 647N (Meissner *et al.* 2018).

### FISH protocol

FISH labeling followed the protocol of Long *et al.* (2017) with the following modifications, unless otherwise specified. After initial dehydration (and associated tissue shrinkage), samples were mounted in 75% ethanol on poly-l-lysine-coated coverslips. They were returned to 4° 100% ethanol for storage until beginning the main FISH protocol. One to four coverslips were moved between jars (W900180-6; Wheaton, Millville, NJ) containing 10 ml of solution for most processing steps. Multiple jars were processed in parallel when needed. Hybridization was performed in custom plexiglass chambers modified from https://hhmi.flintbox.com/public/project/26606/ and related designs [Figure 3A, Figure S5A, and Wu *et al.* (2016)]. The 22 × 22-mm coverslip is held above the bottom of the chamber by 0.5 × 5.5 × 22-mm spacers on each side, leaving an ∼0.5 × 11 × 22-mm space for the samples and 150–180 μl of hybridization solution. A hole at the top allows for overflow, and access for coverslip addition and removal. The 20-hr hybridization reaction was carried out with the chambers inside a humidified polypropylene container (2249-6; Ted Pella). FISH probes were labeled with one of the following fluorophores: Cy3 (GE PA13101), Cy5 (GE PA15101), Quasar 570 (LGC Biosearch Technologies), CF594 (92132; Biotium), Alexa Fluor 594 (AF594) (A20004; Thermo Fisher Scientific), DyLight550 (DL550) (62262; Thermo Fisher Scientific), or CAL Fluor 610 Red (LGC Biosearch Technologies). Please see Supplemental Material for step-by-step coverslip FISH protocol. For serotonin immunostaining with FISH, the tissues were first exposed to 1:50 mouse anti-serotonin (MS-1431-S0; Thermo Fisher Scientific) overnight at 4°. After washing the primary antibody, brain tissues were prepared for FISH. Next, 1:400 AF568 goat anti-mouse secondary (A-11031; Thermo Fisher Scientific) was added in the second step of hybridization and incubated along with FISH probes. After the series of wash steps described in the FISH protocol, the tissues were fixed and mounted with distyrene, plasticizer, and xylene (DPX).

### Confocal imaging

Samples were imaged on Zeiss ([Carl Zeiss], Thornwood, NY) LSM 710, 780, or 880 confocal microscopes and Zeiss ZEN software. Excitation and detection bands for each dye were generally as follows: AF488 with a 488-nm laser and 498–543-nm detection, DL550 and Cy3 with a 561-nm laser and 569–595-nm detection, CF594 with a 594-nm laser and 600–638-nm detection, and ATTO 647N and Cy5 with a 633-nm laser and 638–735-nm detection. Due to microscope limitations, most four-color images were captured as two separate image stacks, the first with 488- and 594-nm channels, and the second with 488-, 561-, and 633-nm channels. The two stacks were merged after imaging for further analysis. Confocal parameters for each image were individually optimized for signal quality unless stated otherwise. All confocal images are full maximum-intensity projections unless stated otherwise. Images were processed with Fiji software (Schindelin *et al.* 2012).

### FISH probe sequences

Probe sequences are listed in Table S1.

### Data availability

All fly strains are available in the Bloomington *Drosophila* Stock Center (http://flystocks.bio.indiana.edu) or upon request. FISH probe sequences are listed in Table S1. All data necessary for confirming the conclusions of the article are present within the article and figures. Supplemental material available at Figshare: https://doi.org/10.25386/genetics.7455137.

## Results and Discussion

### Detection of dopaminergic, serotoninergic, tyraminergic, octopaminergic, and histaminergic neurons in the intact *Drosophila* CNS

**Figure 1:**
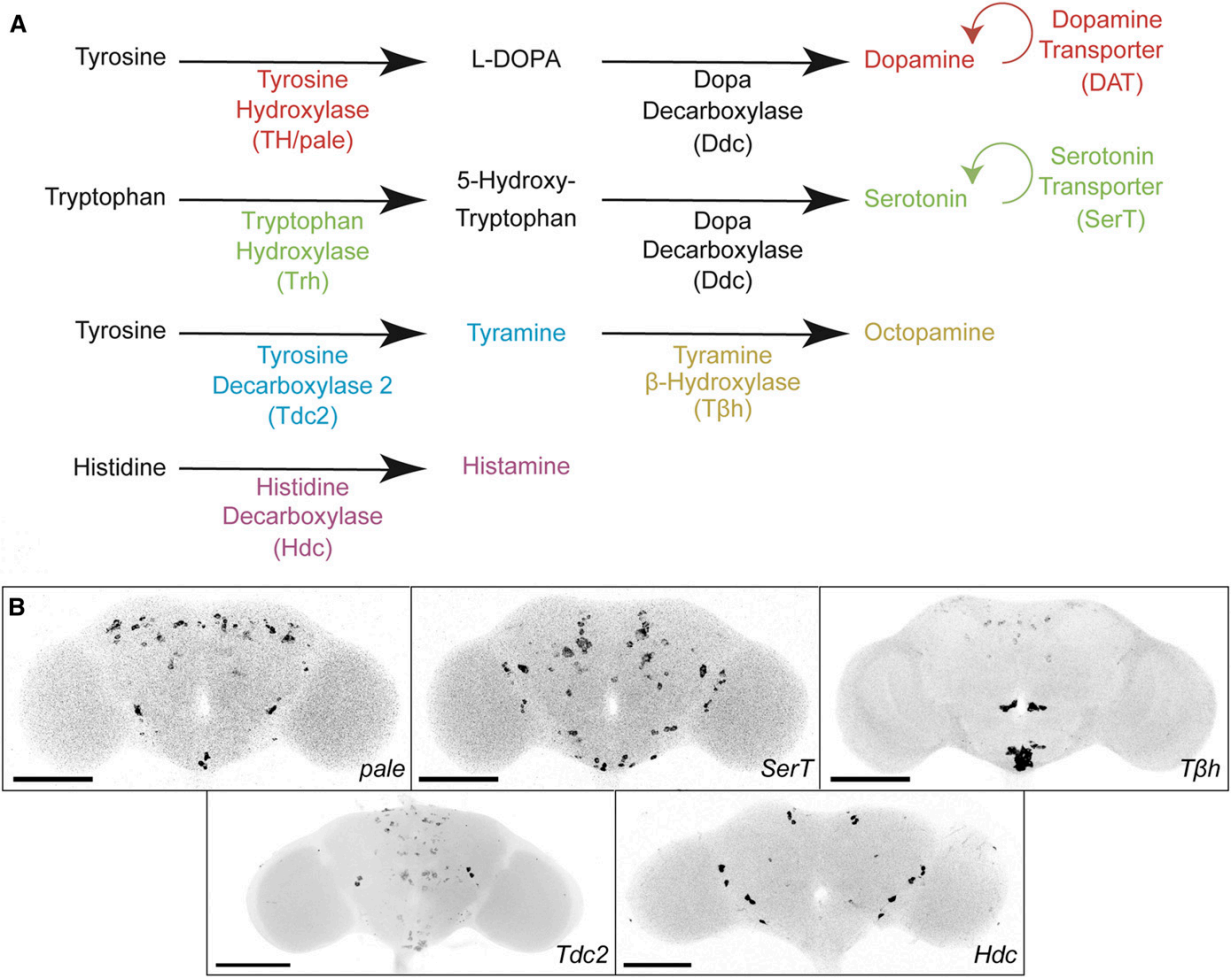
Detection of dopaminergic, serotoninergic, tyraminergic, octopaminergic, and histaminergic neurons in whole-mount *Drosophila* brains using FISH. (A) Biosynthetic pathway of neurotransmitters. To detect neurotransmitter-specific cell types, FISH probes were targeted to mRNAs for specific pathways. Colors indicate pairs of targeted neurotransmitters and mRNAs. (B) Confocal images of *TH/pale*, *SerT*, *T*β*h*, *Tdc2*, and *Hdc* expression patterns in the brain. CNS images of these samples are in Figure S1. Samples were imaged with a 20× objective and intensity values were inverted. Bar, 100 μm.

We previously reported the identification of cholinergic, glutamatergic, and GABAergic neurons in whole-mount *Drosophila* brains by FISH with probes for *Gad1*, *vGlut* and *ChAT* mRNAs (Long *et al.* 2017). This method was based on hybridizing mRNA to multiple short, singly labeled oligonucleotides, combined with tissue-specific treatments for improved FISH in the *Drosophila* brain. To expand this approach to additional neurotransmitters, we developed and validated FISH probes to detect dopaminergic, serotoninergic, octopaminergic, tyraminergic, and histaminergic neurons by probing mRNAs that are specific to their synthesis or transport (Figure 1).

**Figure 2:**
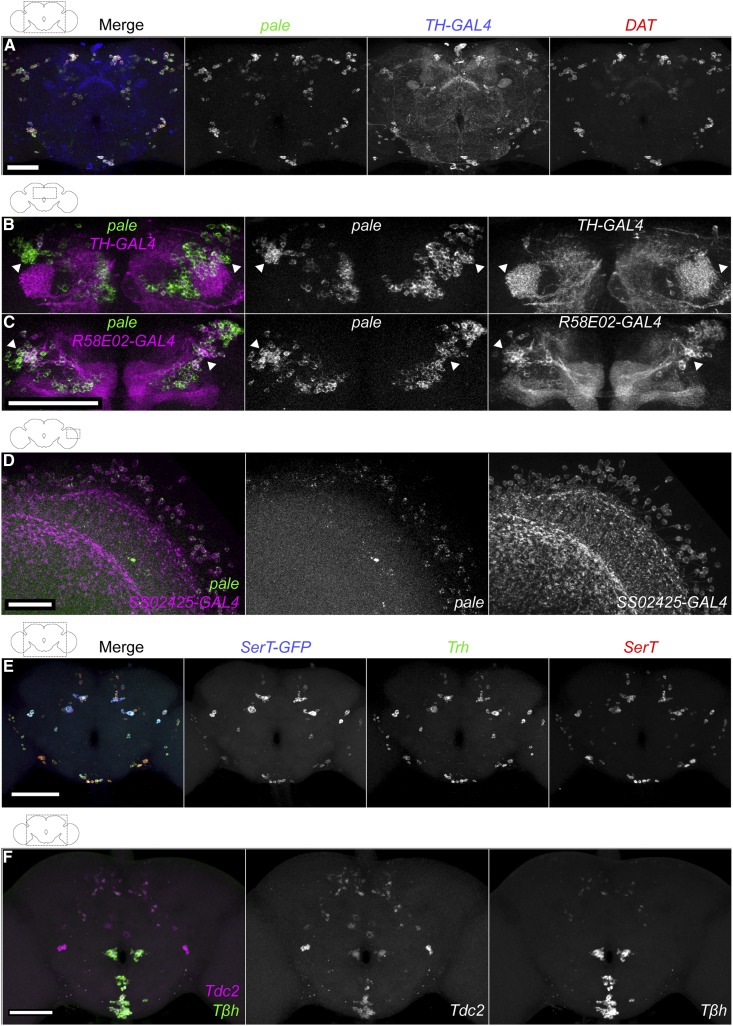
Validation of FISH probes for identification of dopaminergic, serotoninergic, tyraminergic, and octopaminergic neurons. (A) Simultaneous detection of *pale* and *DAT* mRNAs with *TH-GAL4*. A *TH-GAL4*; *UAS-HaloTag* brain was labeled with ATTO 647N HaloTag ligand (blue), and FISH probes for *pale* (Quasar 570; green) and *DAT* (CAL Fluor 610 Red; magenta) mRNAs, shown together (left) and as individual grayscale images. Samples were processed using microcentrifuge protocol. Bar, 50 μm. (B and C) *pale* and GAL4 marker expression in the dorsal anterior brain, focusing on neurons of the protocerebral anterior medial cluster. In *TH-GAL4* (B) and *R58E02-GAL4* (C), *UAS-HaloTag* brains were labeled with AF488 HaloTag ligand (magenta) and *pale* FISH probe (Cy5; green), shown together (left) and as individual grayscale images. Images are maximum intensity z-projections through the anterior central brain. Arrowheads indicate neurons without overlap in (B) and neurons with overlap in (C). Bar, 50 μm. Movies of (B and C) are in Files S8 and S9, respectively. (D) *pale* expression in Mi15 medulla neurons. An *SS02425-GAL4*, *UAS-HaloTag* brain was labeled with ATTO 647N HaloTag ligand (magenta) and *pale* FISH probe (Quasar 570; green) together (left) or as individual grayscale images (Davis *et al.* 2018). Bar, 20 μm. (E) A *SerT-GFP* brain was labeled with FISH probes for *GFP* (AF488; blue), *Trh* (Cy3; green), and *SerT* (Cy5; red) mRNAs. Each channel is shown in gray on the right. Bar, 100 μm. (F) Simultaneous detection of *Tdc2* and *T*β*h* mRNAs. A wild-type brain was labeled with FISH probes for *Tdc2* (Cy5; magenta) and *T*β*h* (Cy3; green). The channels are shown to the right in gray. Bar, 50 μm. Movie is in File S11. The overlap of *Tdc2* and *T*β*h* FISH probes with *Tdc2-GAL4* is shown in Figure S3C.

To identify dopaminergic neurons, we probed *pale* mRNA, encoding tyrosine hydroxylase (TH), a key enzyme for dopamine biosynthesis (Neckameyer and White 1993) (Figure 1A). We also probed *DAT* mRNA, encoding a dopamine transporter that mediates reuptake of dopamine from the synaptic cleft (Penmatsa *et al.* 2013; Wang *et al.* 2015). To validate our approach, we compared *pale* and *DAT* expression patterns with a GAL4 reporter line for tyrosine hydroxylase (*TH-GAL4*) expressing the HaloTag protein (Friggi-Grelin *et al.* 2003; Kohl *et al.* 2014; Sutcliffe *et al.* 2017). We observed widespread overlap (Figure 2A), with only a few exceptions, such as a small group of cells in the superior medial brain (Figure S2A).

*TH-GAL4* was not observed in the dopaminergic protocerebral anterior medial (PAM) cluster or the medulla region of the optic lobe, but the *pale* probe was present, consistent with previous reports (Budnik and White 1988; Liu *et al.* 2012; Davie *et al.* 2018) (Figure 2B and File S8). On the other hand, lines *R58E02-GAL4* and *SS02425* have been reported to label the PAM cluster and dopaminergic Mi15 neurons in the optic lobe, respectively (Liu *et al.* 2012; Davis *et al.* 2018). We observed extensive colocalization of our *pale* FISH probes with the *R58E02* and *SS02425* driver lines (Figure 2, C and D, and File S9). We identified an average of 723 ± 92 *pale*-expressing neurons in the whole brain, including 257 + 11 across both central brain hemispheres and 218 ± 26 in a single optic lobe (*n* = 4 central brains and 7 optic lobes; count ± SE), consistent with previous reports (Mao and Davis 2009). Most *pale*-positive optic lobe neurons are small and had relatively weak FISH signals, hindering precise counts.

To identify serotoninergic neurons, we probed *SerT* mRNA, encoding a serotonin transporter that returns serotonin from the synaptic cleft to presynaptic neurons (Figure 1A) (Giang *et al.* 2011). To validate the probe, we compared it to three other reporters for serotonin expression: (1) *SerT-GFP*, in which the endogenous *SerT* locus was modified to express a SerT-GFP fusion protein (Venken *et al.* 2011; Nagarkar-Jaiswal *et al.* 2015); (2) a serotonin antibody, which has been reported to show identical cell-type specificity as the SerT protein (Giang *et al.* 2011); and (3) a *Trh* FISH probe. *Trh* mRNA encodes tryptophan hydroxylase, a key enzyme involved in serotonin synthesis (Figure 1A) (Neckameyer and White 1992; Coleman and Neckameyer 2005; Neckameyer *et al.* 2007). We observed consistent colocalization across several combinations of the reporters (Figure 2E and Figure S2, B and C), although the wide range and moderate background of antibody labeling makes its full coexpression less certain. In addition, we identified an average of 98 ± 12 cells in the brain with *SerT* FISH signal (*n* = 6; count ± SE), in agreement with previous reports (Vallés and White 1988). Together with previous work on SerT as a marker for serotoninergic cells (Giang *et al.* 2011), these results indicate that our *SerT* FISH probes specifically identify serotoninergic neurons.

Coexpression of serotonin and tyrosine hydroxylase proteins in PPL1 neurons was recently reported (Niens *et al.* 2017). To determine whether we can also detect these coexpressing neurons, we simultaneously probed *pale* and *SerT* mRNAs. We observed two pairs of neurons with consistent overlapping signals within the PPL1 cluster region (Figure S3A). We also observed *SerT* mRNA overlap with *TH-GAL4/UAS-HaloTag* (Figure S3B).

To identify tyraminergic and octopaminergic neurons, we probed *Tdc2* (Tyrosine decarboxylase 2) and *T*β*h* (Tyramine β hydroxylase) mRNAs. We identified an average of ∼116 ± 7 *Tdc2* and 90 ± 5 *T*β*h* neurons in the brain (*n* = 4–6; count ± SE). We observed *Tdc2* expression in 19.0 ± 1.2 ventral and 13.3 ± 0.7 anterior large cell bodies, along with weakly expressing neurons in the dorsal and posterior brain. We identified *T*β*h* expression in 23.4 ± 2.4 ventral and 10.5 ± 0.5 anterior large cell bodies, along with weakly expressing neurons in the lateral and posterior central brain.

The biosynthetic pathway of octopamine in neurons is controlled by both Tdc2 and Tβh: first Tdc2 converts tyrosine to tyramine, then Tβh converts tyramine to octopamine (Figure 1A). Thus, *Tdc2* is predicted to label both tyraminergic and octopaminergic neurons, whereas *T*β*h* should be specific to octopaminergic neurons. We examined the colocalization of *Tdc2* and *T*β*h* FISH probes with each other and *Tdc2-GAL4/UAS-HaloTag*, which labels many tyraminergic/octopaminergic neurons (Cole *et al.* 2005). We observed extensive overlap in the ventral and anterior brain, and more limited overlap in the dorsal and posterior brain (Figure 2F, Figure S3C, and File S11). Most of the nonoverlapping cells showed *Tdc2* but not *T*β*h* signals, and are therefore presumably tyraminergic, but a few weakly labeled cells appeared to only express *T*β*h*. Cole *et al.* (2005) and Busch *et al.* (2009) also observed inconsistencies between *Tdc2-GAL4* and octopamine immunoreactivity in the dorsal and posterior brain, including octopaminergic neurons not labeled by *Tdc2-GAL4*. Although the biosynthetic pathway would predict that all *T*β*h* neurons also express *Tdc2*, this may not be universally true, although it could also be explained by a difference in timing of expression.

For the identification of histaminergic neurons, we probed *Hdc* mRNA, encoding histidine decarboxylase, which catalyzes the decarboxylation of histidine to form histamine (Figure 1A). The cell bodies of the most common histaminergic neurons in flies, photoreceptor cells, are located outside the brain and were removed during dissection. However, histamine is also present in some central brain neurons. In wild-type flies we identified an average of 20.8 ± 1.4 cells with a *Hdc* FISH signal (*n* = 9; count ± SE), with a distribution similar to previous reports (Figure 1B and Figure S4B) (Nässel 1999). Furthermore, we observed a decrease in *Hdc* FISH signal in *Hdc^JK910^* mutant flies (Figure S4, C and D) (Melzig *et al.* 1996) (Burg *et al.* 1993). Thus, our *Hdc* probe appears to specifically label histaminergic neurons.

Together, these results establish a FISH probe set for major neurotransmitter markers in *Drosophila*. Because all the tested probes showed the expected specificity, this also suggests that mRNA detection with our approach is a reliable tool for cell-type identification based on marker expression in general.

### High-throughput FISH efficiently detects neurotransmitter markers

**Figure 3:**
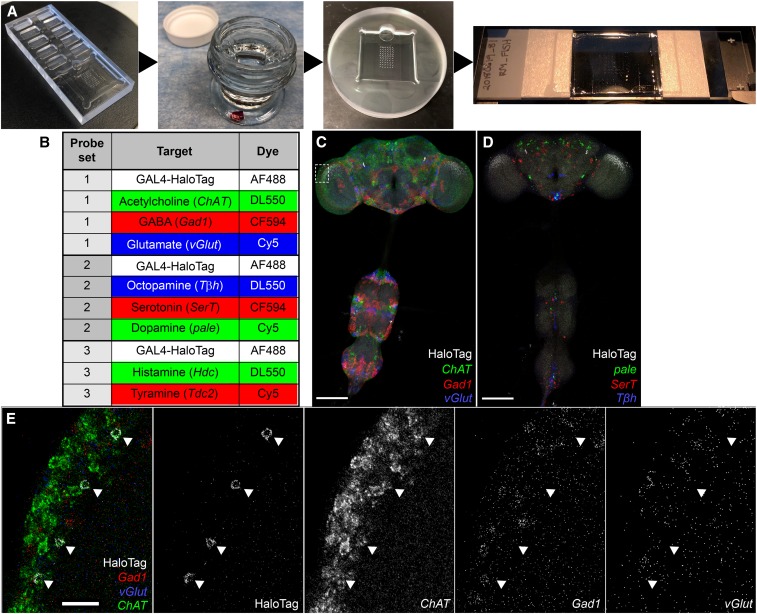
High-throughput FISH platform for identifying neurotransmitter phenotypes in *Drosophila* CNS. (A) Key steps and equipment of the high-throughput FISH platform. Samples are mounted on a coverslip using a plexiglass mounting T-dish (see *Materials and Methods*), using the printed grid beneath the coverslip as a guide. Most processing steps occur by moving coverslips between jars of solution. Hybridization is carried out with the coverslip resting on spacers to either side of a custom hybridization chamber, trapping ∼150 μl of hybridization solution with the samples between the coverslip and the bottom of the chamber. For imaging, the coverslip is mounted in distyrene, plasticizer, and xylene on a slide with a split coverslip for spacers. A schematic of the hybridization chamber is shown in Figure S5A. (B) Neurotransmitter marker detection with optimized FISH probe sets and fluorophore selection. Each set permits detection of two or three FISH probes together with a HaloTag reporter. Movies of optimized probe sets without HaloTag reporter are in Files S1–S6. (C–E) Neurotransmitter detection using the FISH platform. Identifying the neurotransmitter phenotypes of a population of medulla neurons. *SS02565*, *UAS-HaloTag* brains were labeled with AF488 HaloTag ligand (white) and (C) FISH probes for *Gad1* (CF594; red), *vGlut* (Cy5; blue), and *ChAT* (DL550; green) mRNAs or (D) *SerT* (CF594; red), *pale* (Cy5; blue), and *T*β*h* (DL550; green) mRNAs. Bar, 100 μm. (E) Boxed region from (C) was imaged with a 63× objective. Individual channels are shown to the right in gray. Arrowheads indicate the location of HaloTag-labeled cell bodies. Movie is in File S7. Bar, 10 μm.

While our earlier detection of cholinergic, glutamatergic, and GABAergic neurons in a single brain showed the multiplex capability of the described FISH method, the approach is not optimized for processing large numbers of specimens simultaneously (Long *et al.* 2017). To increase throughput, we adapted an approach previously used for large-scale immunostaining, in which fly brains of different genotypes were mounted on a coverslip for parallel labeling (Wu *et al.* 2016). Multiple coverslips, each with up to 60 identified CNSs, can easily be moved between jars for most steps of the FISH process. We created a hybridization chamber with a minimal reaction volume, based on an existing plexiglass mounting dish design (Li *et al.* 2014; Wu *et al.* 2016). The chamber holds the samples in a space of ∼150 μl underneath the coverslip, which rests on spacers above the base of the chamber (Figure 3A). The coverslip processing approach maintains the original FISH procedure but is less laborious than the separate processing of individual brains (Figure S5).

We optimized the fluorophore combination for the neurotransmitter markers used in combination. Figure 3B show the fluorophores for each FISH probe set. The rationale behind this improved scheme is as follows. (1) We reserve the 488-nm laser channel for a cell identity marker, which gives the flexibility to use GFP and derivatives for cell labeling if needed. (2) CF594 works well in DPX-mounted *Drosophila* brain tissues for standard confocal microscopy. It gives a brighter signal and less cross talk than AF594. (3) DL550 performs similarly to Cy3. This gives flexibility to label probes with an alternative fluorophore if the other fluorophore is not available. (4) Fluorophores and FISH probes were paired to balance overall signal levels of each combination. We tested our fluorophore combination using the previously reported FISH probes for *Gad1*, *vGlut* and *ChAT* mRNAs. The similar expression patterns validated the optimized fluorophore combinations (Figure 3C and Files S1 and S2).

With the above optimization, we can simultaneously detect four molecular markers using standard confocal microscopes. To identify the neurotransmitter phenotype for a given cell type, we can rapidly screen through eight different neurotransmitter probes with three parallel FISH experiments. An example is shown in Figure 3C and D. We used a split-GAL4 driver (*SS02565*) to express a HaloTag reporter (*UAS-HaloTag*::*CAAX*) (Sutcliffe *et al.* 2017) in a specific cell population in the optic lobe. HaloTag was labeled with AF488 HaloTag ligand, then samples were colabeled with *Gad1*, *vGlut* and *ChAT*, or *pale*, *Tβh*, and *SerT* FISH probes in two separate experiments with the optimized fluorophore-labeling combination described above. The overlap between AF488 HaloTag ligand and DL550 *ChAT* FISH signals suggests that the neurons of interest are cholinergic, and thus likely to activate their immediate downstream targets (Figure 3C and E). Examples with two additional GAL4 lines are shown in Figure S6.

**Figure 4:**
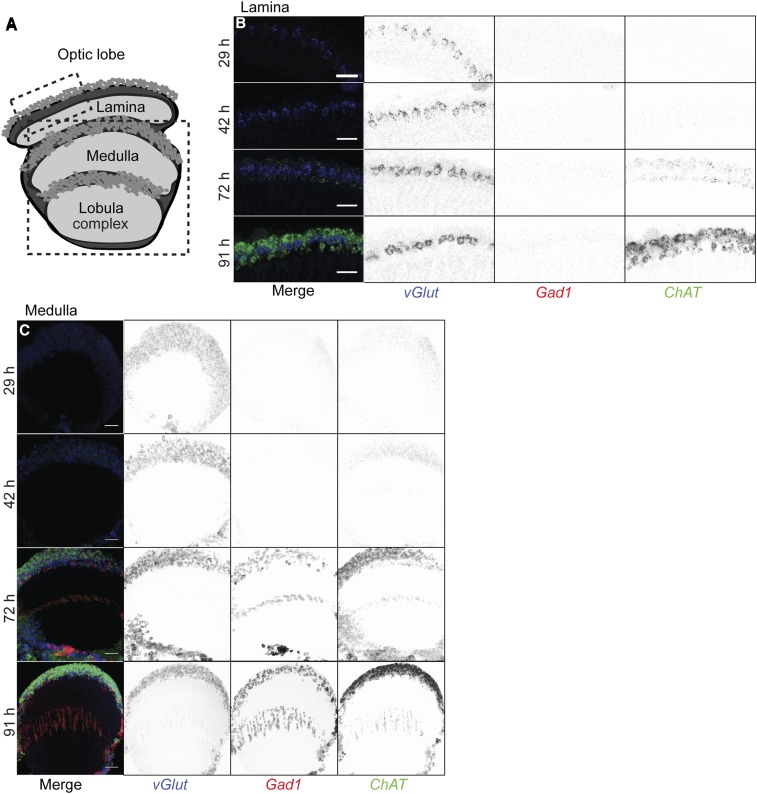
Developmental time course of *vGlut*, *Gad1* and *ChAT* expression in the optic lobe. (A) Schematics of optic lobe subregions. Boxes indicate approximate region of the lamina and medulla imaged. (B and C) Distribution of *vGlut* (Cy5; blue), *Gad1* (CF594; red), and *ChAT* (DL550; green) transcripts in the lamina (B) or medulla (C) at different developmental stages (hours after puparium formation at 25°). Lamina images (B) show single 63× confocal sections. Bar, 20 µm. Medulla images (C) are maximum intensity projections through 25 z-planes for a total depth of 20 µm. Merged images are shown to the left, with split channels inverted in gray to the right. Bar, 10 µm.

While we focused on the adult CNS, this FISH approach is also suitable to investigate neurotransmitter identity in larval and pupal brains. To illustrate the use of FISH for developing neurons, we examined the expression pattern of *Gad1*, *vGlut* and *ChAT* in the visual system at different pupal stages (Figure 4). All three mRNAs were already detectable during the early-to-midpupal stage, in agreement with RNAseq results showing pupal stage *vGlut* and *ChAT* expression in some optic lobe cell types (Tan *et al.* 2015). Interestingly, *vGlut* expression appeared earlier than the other two markers.

### Conclusions

In this study, we introduce a high-throughput, multiplexable FISH platform for the identification of eight different neurotransmitter cell types in the intact *Drosophila* CNS. The ability to identify neurotransmitter types reliably provides pivotal information for the understanding of neural circuit function. That being said, expression of the markers used here does not always indicate the transmitter phenotype of a cell [for example, a neuron might lack a biosynthetic enzyme but acquire a transmitter via uptake from the extracellular space, as reported for some GABAergic neurons in *C. elegans* (Gendrel *et al.* 2016)]. Similarly, other potential transmitters that are less-well characterized in flies [*e.g.*, glycine (Frenkel *et al.* 2017)] are not covered by our probe set. However, these limitations are not specific to our FISH approach and could potentially be addressed in the future by examining additional mRNAs with the same methods.

With the coverslip-processing approach and optimized fluorophore labeling combinations, we can map neurotransmitter markers to specific cell types labeled with *Drosophila* genetic driver lines using standard confocal microscopy. While increasing numbers of neural cell types are being described anatomically, many of their transmitter phenotypes remain unknown. High-resolution images from this work can be used to suggest neurotransmitters within a region of interest, which can then be mapped to specific GAL4-labeled cells by colocalization with FISH. We expect that our FISH protocol for mapping neurotransmitters will find widespread use in *Drosophila* neuroscience studies.

The same FISH approach can be used to study other gene expression patterns in *Drosophila*. This may be of particular utility in the developing brain, where dynamic expression patterns require analyses of many different time points, as illustrated here by capturing early neurotransmitter marker expression in the visual system during development. Finally, our protocols could serve as a basis for further development by research groups interested in the analysis of gene expression in thick tissue sections (> 200 μm), which are comparable in thickness to whole-mount fly brains.

## References

[bib1] Budnik V. White K., 1988 Catecholamine-containing neurons in Drosophila melanogaster: distribution and development. J. Comp. Neurol. 268: 400–413. 10.1002/cne.902680309 3129458

[bib2] Burg M. G. Sarthy P. V. Koliantz G. Pak W. L., 1993 Genetic and molecular-identification of a Drosophila histidine-decarboxylase gene required in photoreceptor transmitter synthesis. EMBO J. 12: 911–919. 10.1002/j.1460-2075.1993.tb05732.x 8096176 PMC413291

[bib3] Busch S. Selcho M. Ito K. Tanimoto H., 2009 A map of octopaminergic neurons in the Drosophila brain. J. Comp. Neurol. 513: 643–667. 10.1002/cne.21966 19235225

[bib4] Buxbaum A. R. Haimovich G. Singer R. H., 2015 In the right place at the right time: visualizing and understanding mRNA localization. Nat. Rev. Mol. Cell Biol. 16: 95–109 (erratum: Nat. Rev. Mol. Cell Biol. 16: 513). 10.1038/nrm3918 25549890 PMC4484810

[bib5] Cole S. H. Carney G. E. McClung C. A. Willard S. S. Taylor B. J., 2005 Two functional but noncomplementing Drosophila tyrosine decarboxylase genes: distinct roles for neural tyramine and octopamine in female fertility. J. Biol. Chem. 280: 14948–14955. 10.1074/jbc.M414197200 15691831

[bib6] Coleman C. M. Neckameyer W. S., 2005 Serotonin synthesis by two distinct enzymes in Drosophila melanogaster. Arch. Insect Biochem. Physiol. 59: 12–31. 10.1002/arch.20050 15822093

[bib7] Croset V. Treiber C. D. Waddell S., 2018 Cellular diversity in the Drosophila midbrain revealed by single-cell transcriptomics. Elife 7: e34550. 10.7554/eLife.34550 PMC5927767 29671739

[bib8] Davie K. Janssens J. Koldere D. De Waegeneer M. Pech U., 2018 A single-cell transcriptome atlas of the aging Drosophila brain. Cell 174: 982–998.e20. 10.1016/j.cell.2018.05.057 29909982 PMC6086935

[bib9] Davis F. P. Nern A. Picard S. Reiser M. B. Rubin G. M., 2018 A genetic, genomic, and computational resource for exploring neural circuit function. bioRxiv. Available at https//.org/10.1101/385476. 10.1101/385476 PMC7034979 31939737

[bib10] Dionne H. Hibbard K. L. Cavallaro A. Kao J. C. Rubin G. M., 2018 Genetic reagents for making split-GAL4 lines in Drosophila. Genetics 209: 31–35. 10.1534/genetics.118.300682 29535151 PMC5937193

[bib11] Frenkel L. Muraro N. I. Gonzalez A. N. B. Marcora M. S. Bernabo G., 2017 Organization of circadian behavior relies on glycinergic transmission. Cell Rep. 19: 72–85. 10.1016/j.celrep.2017.03.034 28380364

[bib12] Friggi-Grelin F. Coulom H. Meller M. Gomez D. Hirsh J., 2003 Targeted gene expression in Drosophila dopaminergic cells using regulatory sequences from tyrosine hydroxylase. J. Neurobiol. 54: 618–627. 10.1002/neu.10185 12555273

[bib13] Fritschy J. M., 2008 Is my antibody-staining specific? How to deal with pitfalls of immunohistochemistry. Eur. J. Neurosci. 28: 2365–2370. 10.1111/j.1460-9568.2008.06552.x 19087167

[bib14] Gendrel M. Atlas E. G. Hobert O., 2016 A cellular and regulatory map of the GABAergic nervous system of C. elegans. Elife 5: e17686. 10.7554/eLife.17686 PMC5065314 27740909

[bib15] Giang T. Ritze Y. Rauchfuss S. Ogueta M. Scholz H., 2011 The serotonin transporter expression in Drosophila melanogaster. J. Neurogenet. 25: 17–26. 10.3109/01677063.2011.553002 21314480

[bib16] Grimm J. B. English B. P. Chen J. J. Slaughter J. P. Zhang Z. J., 2015 A general method to improve fluorophores for live-cell and single-molecule microscopy. Nat. Methods 12: 244–250. 10.1038/nmeth.3256 25599551 PMC4344395

[bib17] Henry G. L. Davis F. P. Picard S. Eddy S. R., 2012 Cell type-specific genomics of Drosophila neurons. Nucleic Acids Res. 40: 9691–9704. 10.1093/nar/gks671 22855560 PMC3479168

[bib18] Kohl J. Ng J. Cachero S. Ciabatti E. Dolan M. J., 2014 Ultrafast tissue staining with chemical tags. Proc. Natl. Acad. Sci. USA 111: E3805–E3814. 10.1073/pnas.1411087111 25157152 PMC4246963

[bib19] Konstantinides N. Rossi A. M. Desplan C., 2015 Common temporal identity factors regulate neuronal diversity in fly ventral nerve cord and mouse retina. Neuron 85: 447–449. 10.1016/j.neuron.2015.01.016 25654249 PMC4489680

[bib20] Lécuyer E. Yoshida H. Parthasarathy N. Alm C. Babak T., 2007 Global analysis of mRNA localization reveals a prominent role in organizing cellular architecture and function. Cell 131: 174–187. 10.1016/j.cell.2007.08.003 17923096

[bib21] Lein E. Borm L. E. Linnarsson S., 2017 The promise of spatial transcriptomics for neuroscience in the era of molecular cell typing. Science 358: 64–69. 10.1126/science.aan6827 28983044

[bib22] Li H. H. Kroll J. R. Lennox S. M. Ogundeyi O. Jeter J., 2014 A GAL4 driver resource for developmental and behavioral studies on the larval CNS of Drosophila. Cell Rep. 8: 897–908. 10.1016/j.celrep.2014.06.065 25088417

[bib23] Liu C. Placais P. Y. Yamagata N. Pfeiffer B. D. Aso Y., 2012 A subset of dopamine neurons signals reward for odour memory in Drosophila. Nature 488: 512–516. 10.1038/nature11304 22810589

[bib24] Long X. Colonell J. Wong A. M. Singer R. H. Lionnet T., 2017 Quantitative mRNA imaging throughout the entire Drosophila brain. Nat. Methods 14: 703–706. 10.1038/nmeth.4309 28581495

[bib25] Luan H. Peabody N. C. Vinson C. R. White B. H., 2006 Refined spatial manipulation of neuronal function by combinatorial restriction of transgene expression. Neuron 52: 425–436. 10.1016/j.neuron.2006.08.028 17088209 PMC1713190

[bib26] Mao Z. Davis R. L., 2009 Eight different types of dopaminergic neurons innervate the Drosophila mushroom body neuropil: anatomical and physiological heterogeneity. Front. Neural Circuits 3: 5 10.3389/neuro.04.005.2009 19597562 PMC2708966

[bib27] Meissner G. W. Grimm J. B. Johnston R. M. Sutcliffe B. Ng J., 2018 Optimization of fluorophores for chemical tagging and immunohistochemistry of Drosophila neurons. PLoS One 13: e0200759 10.1371/journal.pone.0200759 30110347 PMC6093644

[bib28] Melzig J. Buchner S. Wiebel F. Wolf R. Burg M., 1996 Genetic depletion of histamine from the nervous system of Drosophila eliminates specific visual and mechanosensory behavior. J. Comp. Physiol. A Neuroethol. Sens. Neural Behav. Physiol. 179: 763–773. 10.1007/BF00207355 8956497

[bib29] Moffitt J. R. Hao J. Bambah-Mukku D. Lu T. Dulac C., 2016 High-performance multiplexed fluorescence in situ hybridization in culture and tissue with matrix imprinting and clearing. Proc. Natl. Acad. Sci. USA 113: 14456–14461. 10.1073/pnas.1617699113 27911841 PMC5167177

[bib30] Nagarkar-Jaiswal S. Lee P. T. Campbell M. E. Chen K. Anguiano-Zarate S., 2015 A library of MiMICs allows tagging of genes and reversible, spatial and temporal knockdown of proteins in Drosophila. Elife 4: e05338. 10.7554/eLife.05338 PMC4379497 25824290

[bib31] Nässel D. R., 1999 Histamine in the brain of insects: a review. Microsc. Res. Tech. 44: 121–136. 10.1002/(SICI)1097-0029(19990115/01)44:2/3<121::AID-JEMT6>3.0.CO;2-F 10084821

[bib32] Neckameyer W. S. White K., 1992 A single locus encodes both phenylalanine hydroxylase and tryptophan hydroxylase activities in Drosophila. J. Biol. Chem. 267: 4199–4206. 1371286

[bib33] Neckameyer W. S. White K., 1993 Drosophila tyrosine hydroxylase is encoded by the pale locus. J. Neurogenet. 8: 189–199. 10.3109/01677069309083448 8100577

[bib34] Neckameyer W. S. Coleman C. M. Eadie S. Goodwin S. F., 2007 Compartmentalization of neuronal and peripheral serotonin synthesis in Drosophila melanogaster. Genes Brain Behav. 6: 756–769. 10.1111/j.1601-183X.2007.00307.x 17376153

[bib35] Niens J. Reh F. Coban B. Cichewicz K. Eckardt J., 2017 Dopamine modulates serotonin innervation in the Drosophila brain. Front. Syst. Neurosci. 11: 76 10.3389/fnsys.2017.00076 29085286 PMC5650618

[bib36] Penmatsa A. Wang K. H. Gouaux E., 2013 X-ray structure of dopamine transporter elucidates antidepressant mechanism. Nature 503: 85–90. 10.1038/nature12533 24037379 PMC3904663

[bib37] Pfeiffer B. D. Ngo T. T. Hibbard K. L. Murphy C. Jenett A., 2010 Refinement of tools for targeted gene expression in Drosophila. Genetics 186: 735–755. 10.1534/genetics.110.119917 20697123 PMC2942869

[bib38] Raj A. van den Bogaard P. Rifkin S. A. van Oudenaarden A. Tyagi S., 2008 Imaging individual mRNA molecules using multiple singly labeled probes. Nat. Methods 5: 877–879. 10.1038/nmeth.1253 18806792 PMC3126653

[bib39] Schindelin J. Arganda-Carreras I. Frise E. Kaynig V. Longair M., 2012 Fiji: an open-source platform for biological-image analysis. Nat. Methods 9: 676–682. 10.1038/nmeth.2019 22743772 PMC3855844

[bib40] Shah S. Lubeck E. Zhou W. Cai L., 2016 In situ transcription profiling of single cells reveals spatial organization of cells in the mouse hippocampus. Neuron 92: 342–357. 10.1016/j.neuron.2016.10.001 27764670 PMC5087994

[bib41] Sutcliffe B. Ng J. Auer T. O. Pasche M. Benton R., 2017 Second-generation Drosophila chemical tags: sensitivity, versatility, and speed. Genetics 205: 1399–1408. 10.1534/genetics.116.199281 28209589 PMC5378102

[bib42] Tan L. Zhang K. X. Pecot M. Y. Nagarkar-Jaiswal S. Lee P. T., 2015 Ig superfamily ligand and receptor pairs expressed in synaptic partners in Drosophila. Cell 163: 1756–1769. 10.1016/j.cell.2015.11.021 26687360 PMC4804707

[bib43] Tirian L. Dickson B., 2018 The VT GAL4, LexA, and split-GAL4 driver line collections for targeted expression in the Drosophila nervous system. bioRxiv. Available at https//.org/10.1101/198648. 10.1101/198648

[bib44] Vallés A. M. White K., 1988 Serotonin-containing neurons in Drosophila melanogaster: development and distribution. J. Comp. Neurol. 268: 414–428. 10.1002/cne.902680310 3129459

[bib45] Venken K. J. Schulze K. L. Haelterman N. A. Pan H. He Y., 2011 MiMIC: a highly versatile transposon insertion resource for engineering Drosophila melanogaster genes. Nat. Methods 8: 737–743. 10.1038/nmeth.1662 21985007 PMC3191940

[bib46] Wang K. H. Penmatsa A. Gouaux E., 2015 Neurotransmitter and psychostimulant recognition by the dopamine transporter. Nature 521: 322–327. 10.1038/nature14431 25970245 PMC4469479

[bib47] Wu M. Nern A. Williamson W. R. Morimoto M. M. Reiser M. B., 2016 Visual projection neurons in the Drosophila lobula link feature detection to distinct behavioral programs. Elife 5: e21022. 10.7554/eLife.21022 PMC5293491 28029094

[bib48] Yang L. Titlow J. Ennis D. Smith C. Mitchell J., 2017 Single molecule fluorescence in situ hybridisation for quantitating post-transcriptional regulation in Drosophila brains. Methods 126: 166–176. 10.1016/j.ymeth.2017.06.025 28651965 PMC5595163

[bib49] Zhao J. Kilman V. L. Keegan K. P. Peng Y. Emery P., 2003 Drosophila clock can generate ectopic circadian clocks. Cell 113: 755–766. 10.1016/S0092-8674(03)00400-8 12809606

